# Changes in the expression of OCT4 in mouse ovary during estrous cycle

**Published:** 2017-03-15

**Authors:** Narges Bagheripour, Saeed Zavareh, Mohammad Taghi Ghorbanian, Seyed Hassan Paylakhi, Seyed Reza Mohebbi

**Affiliations:** 1 *Department of Cellular and Molecular Biology, School of Biology, Damghan University, Damghan, Iran; *; 2 *Institute of Biological Sciences, Damghan University, Damghan, Iran; *; 3 *Gastroenterology and Liver Diseases* *Research* *Center, Research Institute for Gastroenterology and Liver Diseases, Shahid Beheshti University of Medical Sciences, Tehran, Iran.*

**Keywords:** Estrous cycle, Expression, Mouse, OCT4, Ovarian tissue

## Abstract

The transcriptional factor OCT4 regulates pluripotency of stem cells and has an important role during oocyte growth. Whereas, its role has remained ambiguous in ovarian tissue during reproductive cycle. Therefore, this study was aimed to investigate the expression patterns of OCT4 in mouse ovaries during the normal estrous cycle. Adult National Medical Research Institute mice were classified as proestrous, estrous, metestrous and diestrous on the basis of vaginal smear cytology. Their ovaries were removed and the protein and gene expression levels of OCT4 were assessed using immunohistochemical staining and real-time quantitative reverse-transcription PCR, respectively. Immunohistochemical staining revealed the expression of OCT4 in the cytoplasm of corpus luteum cells. In the follicles, OCT4 was expressed in the cytoplasm of granulosa cells. Furthermore, the gene expression levels of OCT4 was significantly higher in the proestrous phase than in the other phases of the estrous cycle (*p *< 0.05). The results indicated that OCT4 gene expression levels are affected by the cyclic pattern of the estrous cycle.

## Introduction

Ovarian tissue is a multifaceted endocrine gland that produces competent follicles and various substances in response to the cyclic pattern of hypophyseal gonado-trophin secretion.^1^ The various substances are synthesized by ovarian tissue including different hormones, growth factors and cytokines essential for the signaling pathways involved in folliculogenesis or oogenesis.^1^ Changes in hormonal conditions are associated with the increased expression of many hormone-responsive genes within the follicular cells and oocytes.^2^^,^^3^ However, the hormonal control of gene expression throughout folliculogenesis and oocyte growth has not been completely established. The expression of oocyte genes plays critical roles during folliculogenesis, oocyte growth and early embryonic development.^4^^-^^6^ Hormones (e.g. glucocorticoids, androgen and progesterone) that contain nuclear receptors regulate the expression of transcription factors related to the Pit-OCT-Unc-domain family.^7^ Embryonic stem cell-like cells have been found in adult ovaries and the knowledge of these cells has generated a great interest in elucidating the mechanisms involved in folliculogenesis and oogenesis.^1^

The OCT4 is pluripotent stem cell marker located at the center of the transcriptional regulatory hierarchy. It serves key functions in maintaining the undifferentiated state of stem cells and the absence of this marker induces the development of a heterogeneous population of highly specialized daughter cells.^8^ The OCT4 is not only expressed in the oocyte nucleus but also in the oocyte and granulosa cell cytoplasm of growing follicles. This finding suggests that this marker plays critical roles in regulating somatic cell activity and post-fertilization phase.^2^^,^^9^

It has been shown that follicle stimulating hormone (FSH) “proestrous” peak affects follicular growth in the next cycle by triggering stem cell activity in the ovarian surface epithelium. This effect leads to primordial follicle assembly and rapid growth and maturity.^10^^-^^12^ In this regard, Patel *et al*. detected FSH receptors in ovarian stem cells.^13^ Thus, the secretion of hypophyseal gonadotrophins during estrous cycles to regulate folliculogenesis affects the expression of the pluripotent stem cell markers. Hence, this study was aimed to investigate whether or not changes in hormonal profile within the different phases of the mouse normal estrous cycle alter the expression patterns of OCT4 at the gene and protein levels in ovarian tissue.

## Materials and Methods

**Animals and experimental design. **This study was performed in accordance with the guidelines of the “Care and Use of Laboratory Animals.” Experimental protocols were reviewed and approved by the Ethical Committee of the School of Biology, Damghan University, Damghan, Iran, ensuring compliance with the Declaration of Helsinki as revised in Tokyo 2004.

Adult virgin female National Medical Research Institute mice aged 6 to 8 weeks (n = 24) were obtained from the Pasteur Institute of Iran. The animals were housed in an animal house under controlled humidity and temperature conditions in a 12 hr/12 hr light/darkness cycle with free access to food and water. The animals were classified as proestrous, estrous, metestrous and diestrous on the basis of vaginal cytology (three mice per group)*. *To determine the estrous phase, the cellular composition of vaginal smears and the relative ratio between cell types were used as previously described with some modifications.^14^ In brief, vaginal smears were prepared by inserting the tip of a plastic pipette filled with 10 µL of PBS (Sigma-Aldrich, Dorset, UK) into the vagina and repeatedly pipetting and flushing. The samples were fixed in 100% methanol (Merck, Quebec, Canada) and stained with methylene blue. The smears were then evaluated using a light microscope under 10× and 40× objective lenses (Nikon, Tokyo, Japan). Three types of cells were recognized. The round and nucleated cells were the epithelial cells, the irregular ones without nuclei were the cornified cells and the small round cells were the leukocytes. After that, the mice were sacrificed and their ovaries were removed.

**Histological studies. **The excised ovaries were collected and fixed in 4% paraformaldehyde for 12 hr at 4 ˚C. Subsequently, the samples were dehydrated in ethanol series of ascending concentrations, cleared in xylene and embedded in paraffin wax. Then, 5 μm-thick transverse sections of tissue were obtained and stained with hematoxylin-eosin (H & E). Tissue images were taken with a camera coupled to a light microscope (Nikon).

**Immunohistochemistry. **The embedded paraffin cross sections of ovarian tissues (5 μm thickness) were mounted on poly-L-lysine-coated slides, deparaffinized in xylene and then hydrated in descending grades of ethanol. These sections were washed with distilled water for 6 min. Afterward, the sections were boiled in deionized water supplemented with 0.01 M sodium citrate antigen retrieval solution (pH 6) using a microwave oven at 720, 360 and 180 W in 3 min intervals. After cooling, the sections were washed with Tris buffer saline containing 25% triton-X100 for 5 min and then treated with 10% normal goat serum in PBS for 1 hr to prevent non-specific staining. Subsequently, the sections were incubated over-night at 4 ˚C with primary antibodies for OCT4 (ab18976; anti-OCT4 antibody; Abcam, Cambridge, UK) diluted in PBS and 2% Tween (Abcam) in compliance with the manufacturers’ instructions. For the negative control, the primary antibody was omitted and the samples were washed thrice with PBS at 5 min intervals. The sections were incubated with goat anti-rabbit IgG fluorescein-conjugated secondary antibodies (FITC, AP132F, Chemocon, Temecula, USA) at 37 ˚C for 1 hr and then washed with PBS for 5 min. Finally, the sections were mounted with Antifade Vectashield mounting medium (Vector Laboratories Ltd.**, **Peterborough, UK) containing 4′,6-diamidino-2-phenylindole and visualized under a ﬂuorescent microscope (E600; Nikon). Analysis was performed independently by two investigators blinded to the identity of the slides. 

**The RNA extraction, reverse transcription and quantitative real-time PCR. **Total RNA of ovarian tissue was extracted using an RNeasy plus mini kit (Qiagen, Valencia, USA) in accordance with the manufacturer’s instructions. The RNA quality was evaluated using the density ratio of 28S to 18S rRNA bands. Then, 1 μg of total RNA was subjected to reverse transcription using random hexamer primers (S0142; Fermentas, Maryland, USA), RevertAid M-Mul V reverse transcriptase (EP0441; Fermentas), Ribolock RNase inhibitor, dNTP mix (R0191; Fermentas ), 5X reaction buffer for reverse transcription (Fermentas) and RNase-free water in accordance with the manufacturer’s instructions for reverse-transcription PCR (RT-PCR). The reaction was carried out in a thermocycler (Mastercycler Gradient; Eppendorf, San Francisco, USA). The thermal profile was consisted of two cycles of 65 ˚C for 5 min and one cycle of 42 ˚C for 60 min.

Real-time PCR was conducted on a Rotor-Gene 6000 machine (Corbett Life Science, Corbett, USA) using EvaGree qPCR MixPlus (SolisBiodyne, Tartu, Estonia). The sequences of the primers used are listed in Table 1. The thermal profile was consisted of an initial denaturation at 94 ˚C for 10 min followed by 40 cycles of denaturation at 94 ˚C for 15 sec, annealing at 58 ˚C for 25 sec and extension at 72 ˚C for 25 sec. Cycle of threshold (Ct) values was assessed using Rotor-Gene software. The expression data of OCT4 were normalized to ACTIN-B as the internal control. The 2^–ΔΔCt^ method was adopted to evaluate the results of real-time RT-PCR. 

**Statistical Analysis. **Statistical analysis was carried out using SPSS version 16 software (SPSS Inc., Chicago, USA). Data were statistically analyzed by one-way ANOVA. Fisher’s least significant difference (LSD) was used as post-hoc test. Significant differences were considered at the *p* < 0.05 level. 

**Table 1:** Oligonucleotide primer sequences for real-time PCR

**Primer**	**Sequence**	**Primer size**	**Product size**
**OCT4-F**	5- CGGAAGAGAAAGCGAACTAGC-3	21	107
**OCT4-R**	5- ATTGGCGATGTGAGTGATCTG-3	21	-
**ACTIN-B-F**	5- GATTACTGCTCTGGCTCCTAG-3	21	147
**ACTIN-B-R**	5- GACTCATCGTACTCCTGCTTG-3	21	-

## Results

**Fig. 1:**
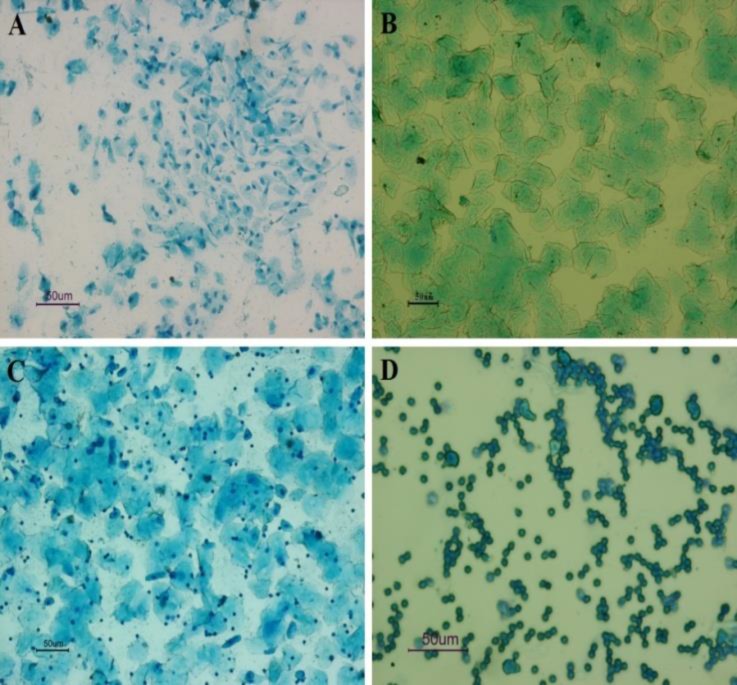
Photomicrographs of methylene blue stained vaginal smears from mice at different phases of estrous cycle; **A)** Proestrous, mostly comprising of nucleated epithelial cells, **B)** Estrus, with predominantly anucleated cornified cells, **C)** Metestrous, containing leukocytes, cornified and nucleated epithelial cells and **D)** Diestrus, comprising largely of leucocytes

**Vaginal cytology. **The cytological appearances of the cells recovered from mouse vaginal washing were corresponded to the four stages of estrous cycle (Fig. 1).

At the proestrous stage, the vaginal opening was reddish pink, swollen and wet. The vaginal smears showed few cornified epithelial cells and few leukocytes with dominant nucleated epithelial cells (Fig. 1A). At the estrous phase, the vaginal opening became lighter pink, less wet and less engorged and the cytological appearance of the vaginal smear showed predominantly anucleated cornified epithelial cells (Fig. 1B). At the metestrous stage, the vaginal opening with pale and unswollen appearance was not open. Furthermore, equal proportions of leukocytes and cornified and nucleated epithelial cells were detected in the vaginal smears (Fig. 1C). At the diestrous stage, the vaginal orifice was small and closed without swelling. Furthermore, a high proportion of leukocytes, some nucleated epithelial cells and mucus were observed in the vaginal smears at this stage (Fig. 1D).

**Fig. 2:**
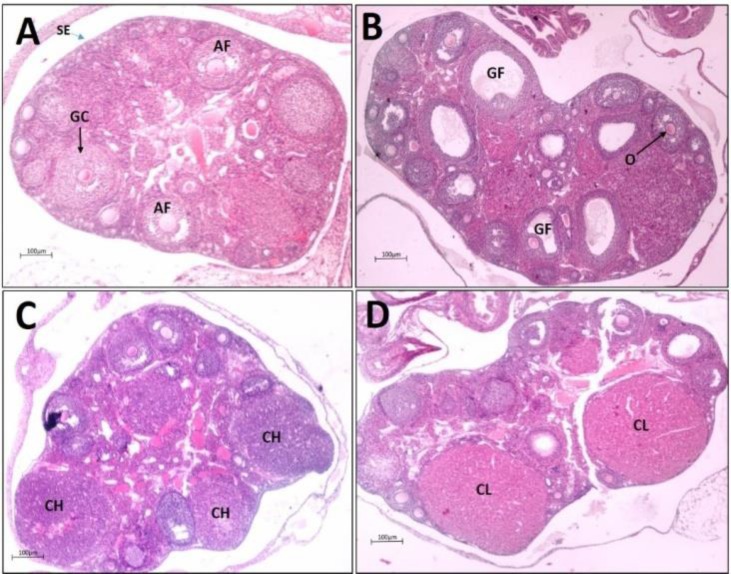
Hematoxylin-eosin stained cross section of ovary from mice at different phases of estrous phase: **A)** Pro-estrous, **B)** Estrous, **C)** Metestrous and **D)** Diestrous. AF: Antral follicle, GC: Granulosa Cell, O: Oocyte, GF: Graffian follicle, CH: Corpus hemorrhagic and CL: Corpus luteum

**Histology study. **Histological findings of the H & E-stained ovarian sections at each stage of estrous cycle are shown in Figure 2. Ovarian sections at the proestrous stage showed degenerated corpora luteum with obvious luteal cell vacuolation and fibrous tissue proliferation. Moreover, several tertiary follicles were noted at this stage. In estrous phase, several noticeable Graafian (preovulatory) follicles with freely floating oocytes were observed. In the metestrous samples, newly formed corpora lutea were detected. These bodies were characterized by small cells with eosinophilic cytoplasm and without cytoplasmic vacuolation and fibrous tissue proliferation. At the diestrous stage, the corpora lutea were appeared in maximum size but with small cytoplasmic vacuoles partially noted within the luteal cells with some fibrous tissue proliferation. Small follicles were also present at this stage.

**Fig. 3:**
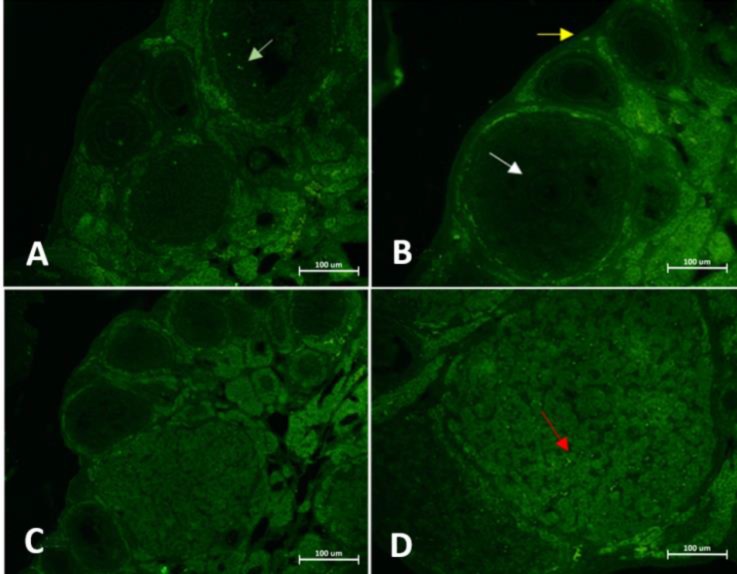
Expression of OCT4 proteins in the ovarian tissue section from mice at different stages of sterous cycle. **A)** Proestrous, **B)** Estrous, **C)** Metestrous, and **D)** Diestrous. Cytoplasm of oocyte (white arrow), granulosa cells (green arrow), luteal cells (red arrow) and stromal cell (yellow arrow

**Immunohistochemical study. **Immunohistochemical results showed that OCT4-specific staining was detectable in the cytoplasm of the mural granulosa cells of the preovulatory follicles. Also, the OCT4 proteins were ubiquitously expressed in the cytoplasm of luteal cells. Furthermore, the cytoplasm of the ovarian stromal cells in all stages of estrous cycle was immunopositive for the OCT4 proteins (Fig. 3). 

**Fig. 4:**
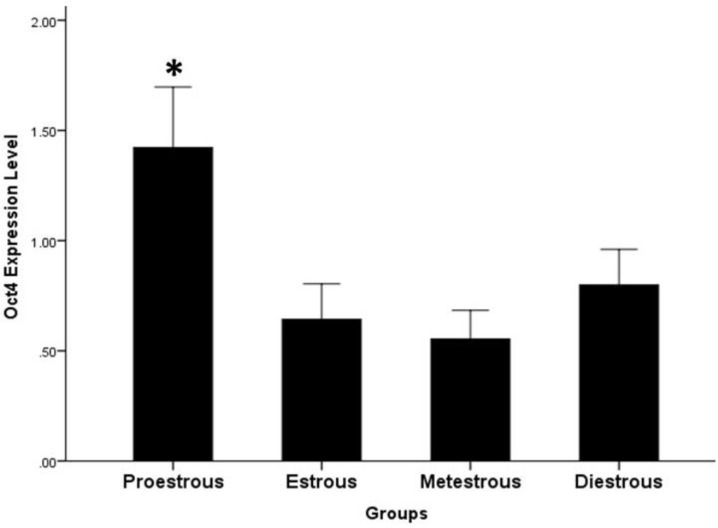
Real-time PCR results of expression of OCT4 in different phases of estrous cycle. * Asterisk indicates expression level significantly higher than other groups (*p *< 0.05

**Real-Time RT-PCR. **The mRNA expression levels of OCT4 were detected in ovarian tissues at all stages of the estrous cycle. Meanwhile, the expression levels of OCT4 were significantly higher in the proestrous phase than in the other phases of the estrous cycle (*p* < 0.05), (Fig. 4). 

## Discussion

The OCT4 is a key regulator of the pluripotency and self-renewal of embryonic stem cells.^15^ The expression levels of OCT4 may regulate neo-oogenesis and folliculo-genesis in some mature ovarian somatic cells.^12^ Moreover, OCT4 expression is related to the onset of meiosis in germ cells because of its protection of apoptosis.^9^ Its expression is reduced in the germinal cell lines from the onset of meiosis to the arrest in prophase I meiotic division and primordial follicle formation. The downregulation of OCT4 expression in germ cells associated with the onset of meiosis may indicate the factors involved in the acquisition of meiotic competence.^16^^,^^17^

The results of present study showed that OCT4 expression is higher in the proestrous stage than in the other stages of the estrous cycle. In the reproductive cycle of adult mice, the FSH peak occurs in the proestrous phase.^18^ It was shown that FSH proliferates the stem cells and germ cell nest formation accompanying with upregulation of pluripotent markers OCT4A, OCT4 and Sox2. Thus, there is a functional interaction between FSH and stem cells in the ovary. Also, an obvious effect of FSH was detected on the ovarian surface epithelium in *in vitro* culture condition.^11^^,^^12^ It was demonstrated that FSH, via its receptor, promotes the proliferation and differentiation of ovarian stem cells.^12^ Thus, the proliferation of ovarian stem cells could be one cause of the upregulation of OCT4 expression in the proestrous phase. Bhartiya *et al*. demonstrated the expression of the R3 type of FSH receptor in the stem cells of ovarian surface epithelium.^11^

Therefore, ovarian stem cells may be regulated by the intercycle FSH peak. Other investigations revealed that FSH not only increases OCT4 expression but also augments the expression of NANOG, survivin and ACT while inhibiting apoptosis. The ACT–survivin signaling pathways have been concluded to possibly inhibit apoptosis in ovarian cancer cells through OCT4 expression by FSH stimulation.^19^

The mechanism of follicle escape from apoptosis up to ovulation remains unclear to date. It seems that FSH and OCT4 exhibit similar effects on the inhibition of follicular cell apoptosis. The activation of anti-apoptotic pathways in the granulosa cells of the dominant follicle is related to OCT4 and FSH expression. 

As a whole, high level of secreted FSH occurs at preovulatory time, nevertheless there is another slighter peak at the late luteal phase which is named the “intercycle peak” in humans or the “proestrus peak” in rodents and is considered to be related to the recruitment of follicles for the next cycle.^10^^,^^11^ It was shown that the “proestrus” peak affects development of follicles and inhibits ovulation in the next estrous cycle. It seems that this proestrus peak of FSH in rodents stimulates stem cell activity in the ovarian surface epithelium, which in turn results in primordial follicle formation.^11^^,^^12^ Therefore, the expression levels of OCT4 not only prepare oocytes for fertilization but also regulate ovarian activity.^9^^,^^19^ Meanwhile, OCT4 expression in the dominant follicle is related to the acquisition of developmental competence by oocytes to resume meiosis, fertilization and support of early embryonic development.^2^ Thus, the cyclic expression of OCT4 during the distinct stages of oogenesis and folliculogenesis indicates the multiple roles of OCT4 at different stages of the process.

It was revealed that SCP3, an essential factor of meiotic division, is expressed in adult mouse and human ovaries at the luteal phase.^20^ The onset of meiotic division is associated with the downregulation of OCT4 expression, which could also be the reason behind the decreased OCT4 expression in luteal phase compared to proestrous phase. 

It has been reported that exogenous FSH and luteinizing hormone administration in mice under the normal estrous cycle upregulates OCT4 in ovarian tissue at the beginning of follicular growth (48 hr after FSH injection) and at the resumption of meiotic division in preovulatory antral oocytes (15 hr after human chorionic gonadotropin injection).^2^ Meanwhile, Patel *et al*. observed the expression of FSH-R and OCT4 in the oocytes of primordial follicles upon entrance to the growth phase, whereas the surrounding granulosa cells were remained quiescent.^13^ In contrast, the location of their expression was changed with follicular growth, that is, from the oocyte to the surrounding granulosa cells. Hence, the expression levels of FSH-R and OCT4 in the mature follicle were reduced in the oocytes and increased in the surrounding granulosa cells.^13^ At the onset of follicular growth, OCT4, through induction of STELLA expression and downregulation of FOXJ2 expression, causes appropriate maturation of oocytes, which consequently supports early embryonic development.^17^ Experiments conducted on the granulosa cells of the mature oocyte complexes revealed that the upregulated expression of OCT4 in these cells increases the number of high-quality retrieved oocytes.^21^ These observations suggest that the selection of the antral follicle for ovulation could be associated with elevated OCT4 expression in oocyte and granulosa cells. This concept agrees well with the results of the immunohistochemical analysis of the present study, which revealed the high expression of OCT4 in the granulosa cells of antral follicles in the proestrous phase. In contrast, the massive atresia of antral follicles in the estrous phase that were not selected for ovulation could account for the downregulated expression of OCT4 in the estrous phase than in the proestrous phase.

Overall, the proliferation of ovarian stem cells, the emergence of antral follicles, and the follicular selection for ovulation in the proestrous phase as well as the massive atresia of antral follicles in the estrous phase and the meiosis initiation of germ cells in the luteal phase may all contribute to the higher OCT4 expression in the pro-estrous phase than in the other phases of the estrous cycle. Meanwhile, the cytoplasmic expression of OCT4 in somatic cells was suggested to occur as a reaction to stress conditions.^22^ Hence, the hormonal secretion by interstitial, hilus and corpus luteum cells may be considered as a stress that leads to the cytoplasmic expression of OCT4. Furthermore, the cytoplasmic expression of this factor may regulate the biological pathways of hormone synthesis.

In conclusion, OCT4 is expressed in the somatic cells of mature ovaries and may jointly cooperate in the regulation of cyclical ovarian activity. Therefore, future studies on OCT4 roles determination in the activation of anti-apoptotic pathways in follicles and the regulation of biological pathways of hormone synthesis should be performed. The contribution of OCT4 to the somatic niche of granulosa cells for the regulation of the growth and maturation of oocytes should also be investigated.

## References

[1] Virant-Klun I, Stimpfel M, Skutella T (2012). Stem cells in adult human ovaries: From female fertility to ovarian cancer. Curr Pharm Des.

[2] Monti M, Garagna S, Redi C (2006). Gonadotropins affect Oct-4 gene expression during mouse oocyte growth. Mol Reprod Dev.

[3] Richards JS (1994). Hormonal control of gene expression in the ovary. Endocr Rev.

[4] Gilchrist R, Ritter L, Armstrong D (2004). Oocyte–somatic cell interactions during follicle development in mammals. Anim Reprod Sci.

[5] Pangas SA, Rajkovic A (2006). Transcriptional regulation of early oogenesis: In search of masters. Hum Reprod Update.

[6] Rajkovic A, Pangas SA, Ballow D (2004). NOBOX deficiency disrupts early folliculogenesis and oocyte-specific gene expression. Science.

[7] Préfontaine GG, Walther R, Giffin W (1999). Selective binding of steroid hormone receptors to octamer transcription factors determines transcriptional synergism at the mouse mammary tumor virus promoter. J Biol Chem.

[8] Brehm A, Ohbo K, Scholer H (1997). The carboxy-terminal transactivation domain of Oct-4 acquires cell specificity through the POU domain. Mol Cell Biol.

[9] Kehler J, Tolkunova E, Koschorz B (2004). OCT4 is required for primordial germ cell survival. EMBO Rep.

[10] Rani CS, Moudgal N (1977). Examination of the role of FSH in periovulatory events in the hamster. J Reprod Fertil.

[11] Bhartiya D, Parte S, Patel H (2016). Novel action of FSH on stem cells in adult mammalian ovary induces postnatal oogenesis and primordial follicle assembly. Stem Cells Int.

[12] Hartiya D, Sriraman K, Gunjal P (2012). Gonadotropin treatment augments postnatal oogenesis and primordial follicle assembly in adult mouse ovaries?. J Ovarian Res.

[13] Patel H, Bhartiya D, Parte S (2013). Follicle stimulating hormone modulates ovarian stem cells through alternately spliced receptor variant FSH-R3. J Ovarian Res.

[14] Singletary SJ, Kirsch AJ, Watson J (2005). Lack of correlation of vaginal impedance measurements with hormone levels in the rat. Contemporary topics in laboratory animal science / American Association for Laboratory Animal Science.

[15] Park IH, Zhao R, West JA (2008). Reprogramming of human somatic cells to pluripotency with defined factors. Nature.

[16] Pesce M, Wang X, Wolgemuth DJ (1998). Differential expression of the Oct-4 transcription factor during mouse germ cell differentiation. Mech Dev.

[17] Zuccotti M, Merico V, Sacchi L (2009). Oct-4 regulates the expression of Stella and Foxj2 at the Nanog locus: Implications for the developmental competence of mouse oocytes. Hum Reprod.

[18] McLean AC, Valenzuela N, Fai S (2012). Performing vaginal lavage, crystal violet staining, and vaginal cytological evaluation for mouse estrous cycle staging identification. J Vis Exp.

[19] Zhang Z, Zhu Y, Lai Y (2013). Follicle-stimulating hormone inhibits apoptosis in ovarian cancer cells by regulating the OCT4 stem cell signaling pathway. Int J Oncol.

[20] Zou K, Yuan Z, Yang Z (2009). Production of offspring from a germline stem cell line derived from neonatal ovaries. Nat Cell Biol.

[21] Varras M, Griva T, Kalles V (2012). Markers of stem cells in human ovarian granulosa cells: Is there a clinical significance in ART?. J Ovarian Res.

[22] Wang X, Dai J (2010). Concise review: Isoforms of OCT4 contribute to the confusing diversity in stem cell biology. Stem Cells.

